# One-Year Impact of COVID-19 Lockdown-Related Factors on Cardiovascular Risk and Mental Health: A Population-Based Cohort Study

**DOI:** 10.3390/ijerph19031684

**Published:** 2022-02-01

**Authors:** Emilie Bérard, Samantha Huo Yung Kai, Nicola Coley, Vanina Bongard, Jean Ferrières

**Affiliations:** 1 UMR 1295 CERPOP, INSERM, UPS, Department of Epidemiology, Health Economics and Public Health, Toulouse University Hospital (CHU), University of Toulouse, 31000 Toulouse, France; emilie.berard@univ-tlse3.fr (E.B.); samantha.huo-yung-kai@univ-tlse3.fr (S.H.Y.K.); nicola.coley@inserm.fr (N.C.); vanina.bongard@univ-tlse3.fr (V.B.); 2 Department of Cardiology, Toulouse University Hospital (CHU), 31000 Toulouse, France

**Keywords:** COVID-19, lockdown, anxiety, depression, cardiovascular risk, general population, cohort study, 1-year follow-up, middle-aged adults, older adults

## Abstract

Lockdown measures have obvious psychological impacts, which could, in turn, increase cardiovascular risk. We assessed the association between lockdown-related factors and the worsening of cardiovascular risk, incident anxiety and depression during 12 months’ follow-up. During lockdown (April–May 2020), 534 subjects, aged 50–89 years, were included in the PSYCOV-CV study (NCT04397835) and followed for up to 12 months post-lockdown. We found that participants with symptoms of depression during lockdown were more likely to report increased cardiovascular drug treatment (Odds-Ratio (OR) = 5.08 (1.78–14.5), *p* = 0.002), decreased physical activity (OR = 1.76 (1.10–2.82), *p* = 0.019) and weight gain (OR = 1.85 (1.08–3.17), *p* = 0.024) after lockdown. Moreover, changes in sleep patterns (OR = 2.35 (1.13–4.88), *p* = 0.022) or living in a rural area during lockdown (OR = 1.70 (0.96–3.03, *p* = 0.069) were associated with higher incident depression, whereas a better relationship with one’s partner during lockdown was associated with less incident depression (OR = 0.56 (0.29–1.08), *p* = 0.084). Finally, we found that continuing to work during lockdown in a role requiring in-person contact with the public (such as cashiers, nurses or physicians) was associated with more incident anxiety after lockdown (OR = 3.38 (1.12–10.2), *p* = 0.031). Interestingly, decreased consumption of alcohol during lockdown was associated with less incident anxiety (OR = 0.30 (0.10–0.90), *p* = 0.032). Our study, conducted in a representative sample of an age group at increased risk of both cardiovascular disease and severe COVID-19, increases the understanding of modifiable factors associated with the health impacts of lockdown measures.

## 1. Introduction

In response to the COVID-19 outbreak, the French government imposed a strict 8-week lockdown of the general population in March 2020, in order to limit viral transmission. During this period, there was a noticeable decrease in non-COVID medical consultations, which may have had a negative impact on the management of patients with cardiovascular and other chronic diseases. Furthermore, the lockdown is likely to have had detrimental effects on psychological health [1,2,3,4], which could have an adverse knock-on effect on cardiovascular risk [5,6,7]. It is therefore important to better understand the consequences of lockdown on psychological health, as well as cardiovascular risk factors and disease, all of which are major public health problems [8]. We previously published [1] factors associated with the worsening of cardiovascular risk and anxiety or depression during the COVID-19 lockdown. Notably, subjects living in urban areas during lockdown and continuing to work in a role requiring contact with the public were more likely to report a deterioration in cardiovascular risk factors during lockdown. Furthermore, living in a home with no outdoor area (garden, terrace or balcony), not being fully convinced by the effectiveness of COVID-related preventive measures, feeling socially isolated or having a deteriorating relationship with one’s partner, during lockdown, were all associated with depression or anxiety during lockdown.

To our knowledge, no study has so far reported the consequences of lockdown with a 12-month follow-up in the general population.

We assessed the association between lockdown-related factors and the worsening of cardiovascular risk, incident anxiety and depression during 12-months’ follow-up, in a representative general population-based cohort study of middle-aged and older adults.

## 2. Materials and Methods

### 2.1. Study Population

**Figure 1:**
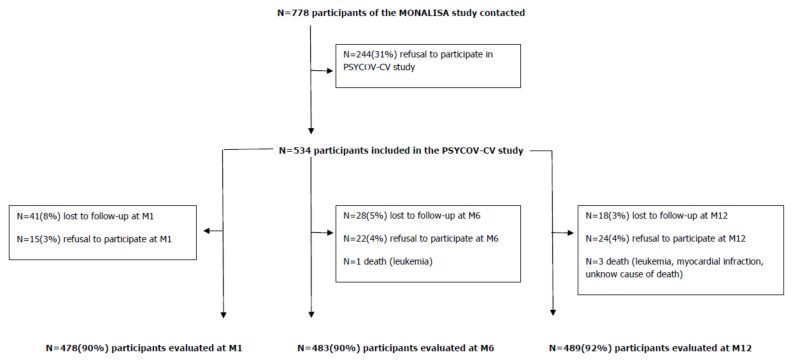
Flow Chart.

A sample of 534 subjects was recruited from the French general population to participate in the PSYCOV-CV cohort study (NCT04397835) during the COVID-19 lockdown (from 17 April 2020 to 10 May 2020). During this period, “the French population was required to stay at home and the only reasons for going out were to go to work (if teleworking was impossible); to do essential food shopping; to travel for health reasons, assisting vulnerable people, family emergencies and childcare; for individual physical activity or to take out a pet (within the limit of one hour per day and within a maximum radius of one kilometre around the home). Any gathering of people who did not live in the same home was prohibited. Infringements to this new rule were penalized initially by a fine of EUR 135 (USD 160), then by a fine of EUR 1500 (USD 1760) in the event of a repeat infringement within 15 days, and in the event of more than 3 infringements within 30 days, the fine was EUR 3750 (USD 4100) and punishable by 6 months imprisonment” [1]. We contacted individuals aged 50–89 years who had previously participated in the south-western France (Toulouse area) MONALISA [9,10] cross-sectional population-based study on the prevalence of cardiovascular risk factors between 2005–2008, to invite them to participate in the PSYCOV-CV cohort study. The participation rate in PSYCOV-CV was 69% (Figure 1). The MONALISA study recruited men and women aged 35–74, and used polling lists, available in each town hall of the survey area, to obtain a stratified random sample. Stratification was applied according to town size (rural versus urban), age and sex, in order to obtain 200 subjects in each sex and 10-year age group (35–44, 45–54, 55–64, 65–74 years). No incentive to participate (in particular no financial incentive) was offered. All subjects gave their informed consent for inclusion before they participated in the MONALISA and PSYCOV-CV study. The MONALISA and PSYCOV-CV studies were conducted in accordance with the Declaration of Helsinki, and the PSYCOV-CV protocol received ethical committee approval in April 2020 (Comité de Protection des Personnes (CPP), Ile de France V, France: protocol code 20.04.03.46101, date of approval 3 April 2020).

### 2.2. Telephone Interviews

Extensive questionnaires were filled in during telephone interviews conducted by trained researchers during lockdown and at 1, 6 and 12 months post-lockdown. The first interview included prospective data (i.e., relating to the lockdown period) and retrospective data concerning the last 2–4 weeks before lockdown. Data concerning socio-economic level, previous personal and family medical history, cardiovascular risk factors, lifestyle habits, drug intake and specific lockdown related factors (such as living in a rural vs. urban environment during lockdown, number of persons living with the participant during lockdown, feelings about lockdown) were recorded. Lipid-lowering, antihypertensive and hypoglycaemic drug use was defined as the current self-reported consumption of a physician-prescribed drug for treating cholesterol, blood pressure or glucose disturbances. Educational level was assessed together with professional activity. Smoking was categorized as regular, occasional (<1 cigarette/day) and non-smoker. Daily consumption of cigarettes and cigarillos were considered for smokers. Alcohol consumption was quantified in glasses per day with a 7-day recall method of a typical week and was categorized into no current consumption, ≥1 glass/week, ≥1 glass/day. Physical activity was assessed in minutes/week for sport (such as walking, bicycling), housework (such as cleaning, tidying, do-it-yourself, gardening, etc.) and in hours/day for TV/computer/smartphone screen time. Height and weight were declared by the participant. Body mass index (BMI) was calculated as weight divided by the square of height in meters (kg/m^2^). Diet quality was recorded using a validated food frequency questionnaire (Supplementary Table S1) [11]. Symptoms of anxiety and depression were recorded using validated scales for the general population (Supplementary Figures S1 and S2): the Generalized Anxiety Disorder-7 (GAD-7: No anxiety: 0–4 points; Mild anxiety: 5–9 points; Moderate anxiety: 10–14 points; Severe anxiety: 15–21 points) and the Patient Health Questionnaire-9 (PHQ-9: No depression: 0–4 points; Mild depression: 5–9 points; Moderate depression: 10–14 points; Moderately severe depression: 15–19 points; Severe depression: 20–27 points) [12,13].

### 2.3. Definition of Endpoints and Analysis Sub-Populations

Worsening of cardiovascular risk was defined as incident:

(i) acute cardiovascular event (ischemic heart disease, atherosclerotic cerebrovascular disease, atherosclerosis in other arteries such as aorta or lower limb arteries, chronic heart failure) during the 12 months after lockdown;
(ii) increased antihypertensive, lipid-lowering or hypoglycaemic physician-prescribed drug treatment (i.e., new or higher post-lockdown dosage prescription compared to pre-lockdown);
(iii) reduced post-lockdown physical activity compared to pre-lockdown (≥15 min/week);
(iv) weight gain (>2 kg) after lockdown (compared to pre-lockdown);
(v) reduction in diet quality (i.e., increased post-lockdown consumption of sugary foods, alcohol, fat or carbohydrates, not compensated by increased fruit and vegetable, dairy (within a limit of 2.5 servings/day), or lean protein consumption); or
(vi) increased smoking (≥1 cigarette/day) during the 12 months after lockdown (compared to pre-lockdown).

Incident anxiety or depression during the 12 months after lockdown were defined, respectively, by a GAD-7 score > 4 or a PHQ-9 score > 4.

In order to consider only incident cases occurring during the 12-month post-lockdown period, participants with worsening of the cardiovascular risk factor of interest during lockdown were excluded from the analysis. Indeed, to assess factors associated with increased physician-prescribed cardiovascular drug treatment (with or without acute cardiovascular event) during the 12 months after lockdown, subjects with increased physician-prescribed cardiovascular drug treatment during lockdown were excluded. Identically, to assess factors associated with a decrease in physical activity after lockdown, subjects with a decrease in physical activity during lockdown were excluded; to assess factors associated with weight gain after lockdown, subjects with weight gain during lockdown were excluded; and to assess factors associated with decrease in diet quality after lockdown, subjects reporting a decrease in diet quality during lockdown were excluded.

Similarly, to assess factors associated with depression after lockdown, subjects with a physician-prescribed depression drug treatment in the 2 weeks preceding lockdown or with depression during lockdown were excluded. Finally, to assess factors associated with anxiety after lockdown, subjects with a physician-prescribed anxiety drug treatment before lockdown or with anxiety during lockdown were excluded.

### 2.4. Statistical Analysis

Statistical analysis was performed using STATA statistical software, release 17.0 (STATA Corporation, College Station, TX, USA). We first described the main characteristics of the participants. The associations between each dependent variable of interest and worsening cardiovascular risk or incident depression and anxiety at up to 12 months after lockdown were first assessed, one by one, in univariate analyses. Then, we assessed lockdown-related factors independently and significantly associated with worsening cardiovascular risk, anxiety and depression using multivariate logistic regression (one multivariate model for each outcome of interest). Variables initially introduced into the multivariate analyses were associated with the endpoint of interest in univariate analyses with a *p*-value < 0.20. A stepwise selection procedure was then applied, sequentially removing variables with a *p*-value > 0.05 from the multivariate model, starting with the variable with the highest *p*-value. When the linearity hypothesis was not respected, continuous variables were transformed into ordinal variables using quartile distributions. Interactions between independent covariates were tested in the final models. None were significant. All reported *p*-values were two-sided and the significance threshold was <0.05.

#### 2.4.1. Worsening Cardiovascular Risk during the 12-Month Post-Lockdown Follow-Up

The links between suspected risk factors observed during lockdown (i.e., lockdown-related factors), particularly anxiety and depression, and worsening cardiovascular risk after lockdown were assessed. Pre-lockdown potential confounding factors (particularly the level of cardiovascular risk factors and the level of anxiety or depression before lockdown) were considered in the analyses, and are described in the Supplementary Table S2 together with the thresholds or categories used in the analyses.

#### 2.4.2. Incident Depression and Anxiety during the 12-Month Post-Lockdown Follow-Up

The links between suspected risk factors observed during lockdown (i.e., lockdown-related factors), particularly worsening cardiovascular risk, and incident depression or anxiety after lockdown were assessed. Pre-lockdown potential confounding factors (particularly the level of cardiovascular risk factors and the level of anxiety or depression before lockdown) were considered in the analyses, and are described in Supplementary Table S2 together with the thresholds or categories used in the analyses.

## 3. Results

### 3.1. Characteristics of the Participants

Of the 534 participants included during lockdown, 48% were men, and the median age was 67 years. All the characteristics of the participants collected at baseline are described in Table 1. Around 90% of the participants were included at each follow-up time (Figure 1).

During the 12-month post-lockdown follow-up period (Table 2), 12% (N = 56) of the participants reported increased antihypertensive, lipid-lowering or hypoglycaemic drug treatment compared to pre-lockdown. Moreover, 65% of the participants reported reduced physical activity [reduction of ≥15 min/week (median reduction = 120 min/week, N = 326), 27% weight gain (>2 kg (median = 3.5 kg), N = 131) and 61% poorer diet quality (i.e., increased consumption of sugary foods, alcohol, fat or carbohydrates, not compensated by increased fruit and vegetable, dairy (within a limit of 2.5 servings/day), or lean protein consumption, N = 304), compared to pre-lockdown. We also observed an increase in smoking (≥1 cigarette/day) in 9% (N = 42) of participants, and eight participants reported acute cardiovascular event (N = 5 ischemic heart disease, N = 2 atherosclerotic cerebrovascular disease and N = 1 atherosclerosis in lower limb arteries). These eight participants also reported increased cardiovascular drug treatment. Furthermore, 35% of participants reported symptoms of depression (Patient Health Questionnaire-9 > 4, N = 172) and 35% symptoms of anxiety (Generalized Anxiety Disorder-7 > 4, N = 175) during the 12-month post-lockdown follow-up.

### 3.2. Determinants of Worsening Cardiovascular Risk during the 12-Month Post-Lockdown Follow-Up

Table 3a shows factors independently and significantly associated with increased physician-prescribed antihypertensive, lipid-lowering or hypoglycaemic drug treatment at up to 12 months after the COVID-19 lockdown (compared to physician-prescribed drug treatments before lockdown). Subjects with increased physician-prescribed cardiovascular drug treatment during lockdown (N = 2 (0.4%)) were excluded. Of note, participants with moderate depression (PHQ-9 ≥ 10) during lockdown were more likely to report an increase in cardiovascular drug treatment after lockdown (Odds-Ratio (OR) = 5.08 (1.78–14.5), *p* = 0.002). Furthermore, smoking more during lockdown was associated with a more frequent increase in cardiovascular drug treatment after lockdown (OR = 5.94 (1.90–18.6), *p* = 0.002) and increased housework (such as cleaning, tidying, do-it-yourself, gardening, etc.) was associated with a less frequent increase in cardiovascular drug treatment after lockdown (OR = 0.42 (0.18–0.96), *p* = 0.039).

Table 3b shows factors independently and significantly associated with a decrease in physical activity (≥15 min/week) at up to 12 months after the COVID-19 lockdown (compared to physical activity before lockdown). Subjects with a decrease in physical activity during lockdown (N = 186 (37.2%)) were excluded. Notably, symptoms of depression (PHQ-9 ≥ 3) or consumption of >3 glasses of alcohol/day during lockdown were associated with a decrease in physical activity after lockdown (OR = 1.76 (1.10–2.82), *p* = 0.019 and OR = 1.49 (0.94–2.38), *p* = 0.093, respectively). Furthermore, smoking less during lockdown was associated with less of a decrease in physical activity after lockdown (OR = 0.29 (0.09–0.98), *p* = 0.046).

Table 3c shows factors independently and significantly associated with weight gain (> 2 kg) at up to 12 months after the COVID-19 lockdown (compared to pre-lockdown weight). Subjects with weight gain >2kg during lockdown (N = 40 (8.1%)) were excluded. In particular, depression (PHQ-9 > 4) during lockdown was associated with weight gain after lockdown (OR = 1.85 (1.08–3.17), *p* = 0.024), as were history of obesity and consumption of ≥1 glass of alcohol/week before lockdown (OR = 2.34 [1.23–4.43], *p* = 0.009 and OR = 2.27 (1.34–3.83), *p* = 0.002, respectively).

Table 3d shows factors independently and significantly associated with decrease in diet quality at up to 12 months after the COVID-19 lockdown (compared to diet quality before lockdown). Subjects reporting a decrease in diet quality during lockdown (N = 138 (27.6%)) were excluded. Of note, participants ≥ 70 years or those who consumed ≥1 glass of alcohol/week during lockdown were more likely to report a decrease in diet quality after lockdown (OR = 1.96 (1.22–3.16), *p* = 0.006 and OR = 1.55 (0.98–2.46), *p* = 0.058, respectively). Furthermore, living with >2 people in the same household during lockdown was associated with less of a decrease in diet quality after lockdown (OR = 0.43 (0.25–0.75), *p* = 0.058).

Table 3e shows factors independently and significantly associated with increased smoking (≥1 cigarette/day) at up to 12 months after the COVID-19 lockdown (compared to smoking before lockdown). After adjustment for increased smoking during lockdown (OR = 14.2 (2.82–71.4), *p* = 0.001), increased alcohol consumption and increased screen time during lockdown were associated with increased smoking after lockdown (OR = 7.52 (2.22–25.5), *p* = 0.001 and OR = 8.34 (2.31–30.1), *p* = 0.001, respectively). Furthermore, those smoking ≥ 3 cigarettes/day before lockdown were less likely to smoke more after lockdown (OR = 0.11 (0.02–0.66) compared to those smoking < 3 cigarettes/day before lockdown, *p* = 0.016).

### 3.3. Determinants of Incident Depression and Anxiety during the 12-Month Post-Lockdown Follow-Up

Table 4a shows factors independently and significantly associated with depression (PHQ-9 > 4) at up to 12 months after the COVID-19 lockdown. Subjects with a physician-prescribed depression drug treatment in the 2 weeks before lockdown or with depression during lockdown (N = 135 (27%)) were excluded. Notably, female gender, and a history of anxiety before or during lockdown were associated with depression after lockdown (OR = 2.96 (1.61–5.43), *p* < 0.001, OR = 5.08 (1.14–22.6), *p* = 0.033 and OR = 2.26 (1.07–4.77), *p* = 0.033, respectively). Furthermore, a change in bedtime/wake-up time ≥ 2 h during lockdown and smoking > 2 cigarettes/day before lockdown were associated with depression after lockdown (OR = 2.35 (1.13–4.88), *p* = 0.022 and OR = 3.24 (1.23–8.53), *p* = 0.017, respectively).

Table 4b shows factors independently and significantly associated with anxiety (GAD-7 > 4) at up to 12 months after the COVID-19 lockdown. Subjects with a physician-prescribed anxiety drug treatment before lockdown or with anxiety during lockdown (N = 132 (26.7%)) were excluded. In particular, participants continuing to work during lockdown in a role requiring in-person contact with the public (such as cashiers, nurses or physicians) were more likely to report anxiety after lockdown (OR = 3.38 (1.12–10.2), *p* = 0.031). Furthermore, decreased alcohol consumption during lockdown was associated with less anxiety after lockdown (OR = 0.30 (0.10–0.90), *p* = 0.032).

## 4. Discussion

We found that participants with symptoms of depression during lockdown were more likely to report an increase in cardiovascular drug treatment, a decrease in physical activity and weight gain after lockdown. Furthermore, as expected, the worsening (or improvement) of cardiovascular risk factors during lockdown was associated with the worsening (or improvement) of cardiovascular risk after lockdown. For example, smoking more during lockdown was associated with higher risk of increased cardiovascular drug treatment (and decreased physical activity) after lockdown and increased physical activity (increased house working or decreased screen time) decreased the risk of increased cardiovascular drug treatment (and decreased the risk of smoking more) after lockdown. Moreover, higher alcohol consumption during lockdown was associated with a decrease in physical activity (or diet quality) and an increase in smoking after lockdown. Finally, life conditions and feelings during lockdown were also associated with the worsening of cardiovascular risk. For example, higher perceived self-risk of COVID-19 infection during lockdown (which is an indicator, amongst other things, of the compliance of the participant to public health advice, and correlated with the adoption of prevention measures, barrier gestures and social distancing) was associated with a lower probability of decreased physical activity (and weight gain) after lockdown. Moreover, having a lifestyle that suited the participant during lockdown was associated with less frequent weight gain after lockdown and living with >2 people (corresponding mostly to a couple with one or more children) during lockdown was associated with less reduction in diet quality after lockdown. Finally, participants who declared that having more time for themselves during lockdown (which they may have used, for example, for cooking) as a positive effect of the lockdown experienced more reduction in diet quality after lockdown, maybe because they abandoned good resolutions after lockdown (indeed, 47% of participants who declared having more time for themselves during lockdown reported increased diet quality during lockdown, compared to 39% of those that did not report having more time for themselves). To our knowledge, our study was the first to prospectively assess the association between lockdown factors (and in particular between depression during lockdown) and the worsening of cardiovascular risk with 12-months’ follow-up in the general population. Nevertheless, in the 5-month follow-up web-based study of Bu et al. (conducted in a non-random sample of adults living in the UK during the COVID-19 pandemic), age, gender, education, income, employment status and physical or mental health were associated with physical activity trajectories (i.e., trajectories with little change over time (62.4%) or with decreasing physical activity over time (28.6%) or with increasing physical activity over time (9%)) [14]. Moreover, Navarro-Pérez et al. [15] used an online cross-sectional questionnaire during the lockdown period and showed that there were no changes in food consumption or routine in over 65 s during lockdown. The authors suggested that this was because this age group has “consolidated habits”. These results are concordant with our previously published data during lockdown (where participants < 60 years were more likely to report a reduction in diet quality during lockdown with OR = 2.17 (1.42–3.32), *p*-value < 0.001) [1]. But here we found that participants ≥ 70 years were more likely to report a reduction in diet quality after lockdown with 12-months’ follow-up.

Concerning depression, as expected [16,17], we found that female gender and history of anxiety were associated with a higher probability of incident depression after lockdown. Interestingly, as Caroppo et al. [18], we found that change in sleep patterns or rural home during lockdown were associated with more incident depression whereas a better relationship with one’s partner during lockdown was associated with less incident depression. This last association is in line with the study of González-Sanguino et al. [19], that explained that spiritual well-being and perceived loneliness were key mental health consequences of the COVID-19 outbreak in Spain. Moreover, Brülhart et al. [20], showed that the peak in helpline calls 6 weeks after the initial outbreak (which offer a real-time measure of mental health concerns) was driven mainly by fear (including fear of infection), loneliness and, later in the pandemic, concerns about physical health.

Concerning anxiety, accordingly with the results of Saunders et al. [17], we found that participants continuing to work during lockdown in a role requiring in-person contact with the public (such as cashiers, nurses or physicians) were more likely to report incident anxiety after lockdown. Furthermore, interestingly, decreased consumption of alcohol during lockdown was associated with less incident anxiety after lockdown.

Our study has several limitations that must be accounted for. First, results are based on declarative data recorded during telephone interviews. Secondly, we may have underestimated cardiovascular events (and the increase in physician-prescribed cardiovascular drugs) due to delayed medical consultations in the COVID-19 context [21]. Cut-offs used for the endpoints may also be criticized. Finally, the study might not be fully representative of the overall French population, as it was drawn from only one French area (south-western France). Despite these limitations, our study is, to our knowledge, the first one to provide a French observational prospective data on the impact on cardiovascular and mental health of the COVID-19 lockdown with 12-months’ follow-up. Moreover, it is a representative study of the general population, aged 50 to 89 years, of the south-western French area. Finally, our sample size (N = 534) exhibits highly clinically and statistically relevant results (considering that randomization was impossible in a study on the impact of the French COVID-19 lockdown that was politically applied nationally), and we adjusted the results for the main confounders.

## 5. Conclusions

In conclusion, we found that modifiable lockdown-related factors were associated, on one hand, with worsening cardiovascular risk and, on the other hand, with incident anxiety and depression after lockdown. We believe that our results increase the understanding of modifiable factors that may be associated with the health impact of the lockdown in a representative sample of people over 50 who are at increased risk of both cardiovascular disease and severe COVID-19, and thus could be subject to additional lockdown periods. 

## Figures and Tables

**Table 1:** Characteristics of the participants before and during the COVID-19 lockdown (17 March 2020 to 10 May 2020, France).

Characteristics	Total N = 534
Age (in years) during lockdown, mean (SD)	66.60 (10.36)
Male gender, *n* (%)	255 (47.8)
Number of persons living with participant—during lockdown, mean (SD)	2.15 (1.12)
Home location—during lockdown	
Urban, *n* (%)	328 (62.2)
Rural, *n* (%)	199 (37.8)
Educational level	
<High school completion, *n* (%)	201 (37.6)
≥High school completion, *n* (%)	321 (60.1)
Other, *n* (%)	12 (2.2)
Professional activity—before lockdown	
Not Working, *n* (%)	11 (2.1)
Working, *n* (%)	203 (38.1)
Retired, *n* (%)	319 (59.8)
Professional activity—during lockdown	
Working (out of home) without in-person contact with the public (e.g., dustmen), *n* (%)	31 (5.8)
Working with in-person contact with the public (e.g., cashiers, nurses), *n* (%)	43 (8.1)
Teleworking, *n* (%)	74 (13.9)
Not working, *n* (%)	386 (72.3)
Change in bedtime/wake-up time ≥ 2 h—during lockdown *, *n* (%)	92 (17.4)
Having more time for oneself **—during lockdown, *n* (%)	147 (31.0)
During the previous 7 days of lockdown, having a lifestyle that suits oneself	
No, *n* (%)	20 (3.8)
Rather No, *n* (%)	40 (7.5)
Rather Yes, *n* (%)	135 (25.4)
Yes, *n* (%)	336 (63.3)
Self-perceived risk of being contaminated by COVID-19 (on a scale from 1 to 10)—during lockdown, mean (SD)	3.73 (2.30)
Estimated relationship with partner (on a scale from 1 to 10)—during lockdown, mean (SD)	8.35 (1.53)
Worsening relationship with partner during lockdown, *n* (%)	33 (8.6)
History of high blood pressure—before lockdown, *n* (%)	169 (31.7)
History of hypercholesterolemia—before lockdown, *n* (%)	128 (24.2)
History of diabetes—before lockdown, *n* (%)	49 (9.2)
Increased antihypertensive, lipid-lowering or hypoglycaemic physician prescribed drug treatment—during lockdown, *n* (%)	2 (0.4)
History of obesity—before lockdown, *n* (%)	80 (15.0)
History of CVD—before lockdown, *n* (%)	64 (12.0)
Family history of premature coronary disease ***—before lockdown, *n* (%)	56 (11.8)
History of anxiety—before lockdown, *n* (%)	157 (29.5)
Anxiety drug treatment—before lockdown, *n* (%)	35 (6.6)
History of depression—before lockdown, *n* (%)	49 (9.2)
Depression drug treatment—before lockdown, *n* (%)	29 (5.4)
Anxiety: GAD-7—during lockdown, mean (SD)	2.80 (3.45)
Anxiety: GAD-7—during lockdown	
No: 0–4 pts, *n* (%)	409 (76.7)
Mild: 5–9 pts, *n* (%)	89 (16.7)
Moderate: 10–14 pts, *n* (%)	28 (5.3)
Severe: 15–21 pts, *n* (%)	7 (1.3)
Depression: PHQ-9—during lockdown, mean (SD)	2.92 (3.31)
Depression: PHQ-9—during lockdown	
No: 0–4 pts, *n* (%)	410 (76.9)
Mild: 5–9 pts, *n* (%)	95 (17.8)
Moderate: 10–14 pts, *n* (%)	23 (4.3)
Moderately severe: 15–19 pts, *n* (%)	5 (0.9)
Smoking—before lockdown	
Yes, regularly (everyday), *n* (%)	50 (9.4)
Yes, occasionally (<1 cig/d), *n* (%)	15 (2.8)
No, *n* (%)	469 (87.8)
Smoking—during lockdown	
Yes, regularly (everyday), *n* (%)	50 (9.7)
Yes, occasionally (<1 cig/d), *n* (%)	7 (1.4)
No, *n* (%)	458 (88.9)
Number of cigarettes/day—before lockdown, mean (SD)	7.43 (6.89)
Number of cigarettes/day—during lockdown, mean (SD)	8.46 (7.75)
Smoking—during lockdown	
Decreased, *n* (%)	17 (3.2)
No change, *n* (%)	496 (92.9)
Increased, *n* (%)	21 (3.9)
Alcohol consumption—before lockdown	
No, *n* (%)	183 (34.3)
≥1 glass/week, *n* (%)	209 (39.1)
≥1 glass/day, *n* (%)	142 (26.6)
Alcohol consumption—during lockdown	
No, *n* (%)	203 (38.0)
≥1 glass/week, *n* (%)	182 (34.1)
≥1 glass/day, *n* (%)	149 (27.9)
Alcohol consumption (glasses/day)—before lockdown, mean (SD)	0.77 (1.20)
Alcohol consumption (in glass/day)—during lockdown, mean (SD)	0.81 (1.63)
Alcohol consumption—during lockdown	
Decreased, *n* (%)	71 (13.3)
No change, *n* (%)	400 (74.9)
Increased, *n* (%)	63 (11.8)
Physical activity (min/week)—before lockdown, mean (SD)	260.54 (322.67)
Physical activity (min/week)—during lockdown, mean (SD)	276.55 (397.64)
Physical activity (min/week)—during lockdown	
Decreased, *n* (%)	193 (36.3)
No change, *n* (%)	154 (28.9)
Increased, *n* (%)	185 (34.8)
Housework **** (min/week)—before lockdown, mean (SD)	236.37 (290.23)
Housework **** (min/week)—during lockdown, mean (SD)	288.33 (363.82)
Housework **** (min/week)—during lockdown	
Decreased, *n* (%)	62 (11.9)
No change, *n* (%)	303 (58.2)
Increased, *n* (%)	156 (29.9)
Increased screen time (hours/day)—during lockdown, *n* (%)	281 (53.1)
Diet quality—during lockdown	
Increased, *n* (%)	133 (28.7)
No change, *n* (%)	188 (40.6)
Decreased *****, *n* (%)	142 (30.7)
Weight gain—during lockdown, *n* (%)	138 (26.4)

* compared to pre-lockdown bedtime/wake-up time. ** As a positive effect of the lockdown felt by the participant. *** Family history of premature myocardial infarction, i.e., before 55 years old for the father or brother and before 65 years old for the mother or sister. **** Such as cleaning, tidying, do-it-yourself, gardening, etc. ***** Decrease in diet quality was defined as in increase in consumption of sugary foods, alcohol, fat or carbohydrates, that was not compensated by increased fruit and vegetable, dairy (within a limit of 2.5 servings/day), or lean protein consumption. CVD: cardiovascular disease (ischemic heart disease, atherosclerotic cerebrovascular disease, atherosclerosis in other arteries such as aorta or lower limb arteries, chronic heart failure). GAD-7: Generalized Anxiety Disorder-7. PHQ-9: Patient Health Questionnaire-9. SD: Standard deviation.

**Table 2:** Worsening of cardiovascular risk and incident depression or anxiety at up to 12 months after the COVID-19 lockdown (17 March 2020 to 10 May 2020, France).

	Total N = 534
Increased physician-prescribed antihypertensive, lipid-lowering or hypoglycaemic drug treatment, *n* (%)	56 (11.5)
Time of increased physician-prescribed antihypertensive, lipid-lowering or hypoglycaemic drug treatment	
M1, *n* (%)	8 (14.3)
M6, *n* (%)	23 (41.1)
M12, *n* (%)	25 (44.6)
Decrease in physical activity (≥15 min/week), *n* (%)	326 (65.1)
Time of decrease in physical activity (≥15 min/week)	
M1, *n* (%)	189 (58.0)
M6, *n* (%)	90 (27.6)
M12, *n* (%)	47 (14.4)
Weight gain > 2 kg, *n* (%)	131 (26.5)
Time of weight gain > 2 kg	
M1, *n* (%)	53 (40.5)
M6, *n* (%)	32 (24.4)
M12, *n* (%)	46 (35.1)
Decrease * in diet quality, *n* (%)	304 (60.8)
Time of decrease in diet quality	
M1, *n* (%)	177 (58.2)
M6, *n* (%)	83 (27.3)
M12, *n* (%)	44 (14.5)
Increased smoking, *n* (%)	42 (8.6)
Time of increased smoking	
M1, *n* (%)	23 (54.8)
M6, *n* (%)	10 (23.8)
M12, *n* (%)	9 (21.4)
Depression (PHQ-9 > 4), *n* (%)	172 (34.6)
Time of depression (PHQ-9 > 4)	
M1, *n* (%)	92 (53.5)
M6, *n* (%)	39 (22.7)
M12, *n* (%)	41 (23.8)
Anxiety (GAD-7 > 4), *n* (%)	175 (35.4)
Time of anxiety (GAD-7 > 4)	
M1, *n* (%)	95 (54.3)
M6, *n* (%)	48 (27.4)
M12, *n* (%)	32 (18.3)

* Decrease in diet quality was defined as increased consumption of sugary foods, alcohol, fat or carbohydrates, that was not compensated by increased fruit and vegetable, dairy (within a limit of 2.5 servings/day), or lean protein consumption. GAD-7: Generalized Anxiety Disorder-7. M1: month 1. M6: month 6. M12: month 12. PHQ-9: Patient Health Questionnaire-9.

**Table 3:** Factors independently and significantly associated with the worsening of cardiovascular risk at up to 12 months after the COVID-19 lockdown (17 March 2020 to 10 May 2020, France) compared to before lockdown—results of stepwise multivariate analyzes.

**a. Factors independently associated with increased antihypertensive, lipid-lowering or hypoglycaemic drug treatment (N = 56/488 ^a^)**		
	**Adjusted ^b^ Odds-Ratio (95% Confidence Interval)**	***p*** **-Value**
Moderate depression (PHQ-9 ≥ 10) ^c^—during lockdown	5.08 (1.78–14.5)	0.002
**Increased smoking** **—during lockdown**	5.94 (1.90–18.6)	0.002
Increased housework ^d^—during lockdown	0.42 (0.18–0.96)	0.039
History of high blood pressure—before lockdown	2.94 (1.56–5.52)	0.001
History of diabetes—before lockdown	3.48 (1.61–7.50)	0.001
**b. Factors independently associated with decrease in physical activity ≥ 15 min/week (N = 161/314 ^e^)**		
	**Adjusted ^b^ Odds-Ratio (95% Confidence Interval)**	***p*** **-Value**
Depression (PHQ-9 ≥ 3 (median)) ^c^—during lockdown	1.76 (1.10–2.82)	0.019
Perceived self-risk of COVID-19 infection (on a scale from 1 to 10) ^f^ ≥ 4 (median)—during lockdown	0.51 (0.32–0.82)	0.006
Decreased smoking—during lockdown	0.29 (0.09–0.98)	0.046
**Increased** **screen time—during lockdown**	1.75 (1.10–2.79)	0.019
Consumption of >3 glasses of alcohol/day—during lockdown	1.49 (0.94–2.38)	0.093
**c. Factors independently associated with weight gain >2 kg (N = 99/451 ^g^)**		
	**Adjusted ^b^ Odds-Ratio (95% Confidence Interval)**	***p*** **-Value**
Depression (PHQ-9 > 4) ^c^—during lockdown	1.85 (1.08–3.17)	0.024
During the last 7 days, lifestyle that didn’t suit the participant—during lockdown	4.29 (1.54–12.0)	0.005
Perceived self-risk of COVID-19 infection (on a scale from 1 to 10) ^f^ > 2 (first quartile)—during lockdown	0.58 (0.35–0.97)	0.038
Weight gain (0–2 kg)—during lockdown	4.31 (2.51–7.40)	<0.001
History of obesity—before lockdown	2.34 (1.23–4.43)	0.009
Consumption ≥ 1 glass of alcohol/week—before lockdown	2.27 (1.34–3.83)	0.002
**d. Factors independently associated with decrease in diet quality ^h^ (N = 201/362 ^i^)**		
	**Adjusted ^b^ Odds-Ratio (95% Confidence Interval)**	***p*** **-Value**
Age ≥ 70 years	1.96 (1.22–3.16)	0.006
Living with >2 people—during lockdown	0.43 (0.25–0.75)	0.003
Having more time for oneself ^j^—during lockdown	2.04 (1.22–3.40)	0.007
Consumption of ≥1 glass of alcohol/week—during lockdown	1.55 (0.98–2.46)	0.058
**e. Factors independently associated with increased smoking ≥1 cigarette/day (N = 42/487)**		
	**Adjusted ^b^ Odds-Ratio (95% Confidence Interval)**	***p*** **-Value**
**Increased smoking** **—during lockdown**	14.2 (2.82–71.4)	0.001
Increased consumption of alcohol—during lockdown	7.52 (2.22–25.5)	0.001
**Increased** **screen time—during lockdown**	8.34 (2.31–30.1)	0.001
Smoking < 3 cigarettes/day—before lockdown (ref)	1.00	
Smoking ≥ 3 cigarettes/day—before lockdown	0.11 (0.02–0.66)	0.016
Non-smokers—before lockdown	0.01 (0.002–0.02)	<0.001

Variables in bold relate to several endpoints for worsening cardiovascular risk. ^a^ Subjects with increased physician-prescribed antihypertensive, lipid-lowering or hypoglycemic drug treatment during lockdown (N = 2 (0.4%)) were excluded, thus leading to an analysis sample of 488 subjects. ^b^ Each Odds-Ratio was adjusted for all the factors shown in the table. ^c^ PHQ-9: Patient Health Questionnaire-9 (No depression: 0–4 points; Mild depression: 5–9 points; Moderate depression: 10–14 points; Moderately severe depression: 15–19 points; Severe depression: 20–27 points). ^d^ Such as cleaning, tidying, do-it-yourself, gardening, etc. (minutes/week). ^e^ Subjects reporting a decrease in physical activity during lockdown (N = 186 (37.2%)) were excluded, thus leading to an analysis sample of 314 subjects. ^f^ Indicator correlated with the adoption of prevention measures, barrier gestures and social distancing according to Santé Publique France, the National Public Health Agency. ^g^ Subjects with weight gain > 2 kg during lockdown (N = 40 (8.1%)) were excluded, thus leading to an analysis sample of 451 subjects. ^h^ Decrease in diet quality defined as increased consumption of sugary foods, alcohol, fat or carbohydrates, that was not compensated by increased fruit and vegetable, dairy (within a limit of 2.5 servings/day) or lean protein consumption. ^i^ Subjects with a decrease in diet quality during lockdown (N = 138 (27.6%)) were excluded, thus leading to an analysis sample of 362 subjects. ^j^ As a positive effect of the lockdown felt by the participant. Ref: reference (Odds-Ratio = 1.00).

**Table 4:** Factors independently and significantly associated with depression (Patient Health Questionnaire-9 > 4) and anxiety (Generalized Anxiety Disorder-7 > 4) at up to 12 months after the COVID-19 lockdown (17 March 2020 to 10 May 2020, France)—results of stepwise multivariate analyzes.

**a. Factors independently associated with depression (PHQ-9 > 4) (N = 79/362 ^a^)**		
	**Adjusted ^b^ Odds-Ratio (95% Confidence Interval)**	***p*** **-Value**
Female gender	2.96 (1.61–5.43)	<0.001
Anxiety (GAD-7 > 4) ^c^—during lockdown	2.26 (1.07–4.77)	0.033
Change in bedtime/wake-up time ≥ 2 h—during lockdown	2.35 (1.13–4.88)	0.022
Living in a rural area—during lockdown	1.70 (0.96–3.03)	0.069
Estimated relationship with partner (on a scale from 1 to 10) > 8 (median)—during lockdown	0.56 (0.29–1.08)	0.084
Symptoms of depression (PHQ-9 = (2–4)) ^d^—during lockdown	1.65 (0.93–2.93)	0.084
Smoking > 2 cigarettes/day ^e^—before lockdown	3.24 (1.23–8.53)	0.017
Physician-prescribed anxiety drug treatment—before lockdown	5.08 (1.14–22.6)	0.033
History of CVD—before lockdown	2.09 (0.88–4.99)	0.096
**b. Factors independently associated with anxiety (GAD-7 > 4) (N = 83/363 ^f^)**		
	**Adjusted ^b^ Odds-Ratio (95% Confidence Interval)**	***p*** **-Value**
Job (out of home) without in-person contact with the public during lockdown (e.g., dustmen) or teleworking (ref)	1.00	
Job with in-person contact with the public during lockdown (e.g., cashiers, nurses)	3.38 (1.12–10.2)	0.031
No job during lockdown	1.92 (0.89–4.13)	0.096
Anxiety (for each point of GAD-7) ^c^—during lockdown	1.91 (1.56–2.34)	<0.001
Decreased alcohol consumption—during lockdown	0.30 (0.10–0.90)	0.032

^a^ Subjects with a physician-prescribed depression drug treatment before lockdown or with depression (PHQ-9 score >4) during lockdown (N = 135 (27%) were excluded, thus leading to an analysis sample of 362 subjects. ^b^ Each Odds-Ratio was adjusted for all the factors shown in the table. ^c^ GAD-7: Generalized Anxiety Disorder-7 (No anxiety: 0–4 points; Mild anxiety: 5–9 points; Moderate anxiety: 10–14 points; Severe anxiety: 15–21 points). ^d^ PHQ-9: Patient Health Questionnaire-9 (No depression: 0–4 points; Mild depression: 5–9 points; Moderate depression: 10–14 points; Moderately severe depression: 15–19 points; Severe depression: 20–27 points). ^e^ Compared to non-smokers or smoking ≤ 2 cigarettes/day—before lockdown. ^f^ Subjects with a physician-prescribed anxiety drug treatment before lockdown or with anxiety (GAD-7 score > 4) during lockdown (N = 132 (26.7%)) were excluded, thus leading to an analysis sample of 363 subjects. CVD: cardiovascular disease (ischemic heart disease, atherosclerotic cerebrovascular disease, atherosclerosis in other arteries such as aorta or lower limb arteries, chronic heart failure).

## Data Availability

Not applicable.

## Supplementary Materials

The following are available online at https://www.mdpi.com/article/10.3390/ijerph19031684/s1, Table S1: Food frequency questionnaire, Table S2: Suspected risk factors and potential confounders (dependent variables) assessed (one by one) in univariate analyses for worsening cardiovascular risk, incident depression and anxiety outcomes during the 12-month post-lockdown follow-up, Figure S1: Generalized Anxiety Disorder-7, Figure S2: Patient Health Questionnaire-9.

## References

[1] Bérard E., Kai S.H.Y., Coley N., Bongard V., Ferrières J. (2021). Lockdown-related factors associated with the worsening of cardiovascular risk and anxiety or depression during the COVID-19 pandemic. Prev. Med. Rep..

[2] Brooks S.K., Webster R., Smith L.E., Woodland L., Wessely S., Greenberg N., Rubin G.J. (2020). The psychological impact of quarantine and how to reduce it: Rapid review of the evidence. Lancet.

[3] Luykx J.J., Vinkers C.H., Tijdink J.K. (2020). Psychiatry in Times of the Coronavirus Disease 2019 (COVID-19) Pandemic: An Imperative for Psychiatrists to Act Now. JAMA Psychiatry.

[4] Richter D., Riedel-Heller S., Zürcher S.J. (2021). Mental health problems in the general population during and after the first lockdown phase due to the SARS-CoV-2 pandemic: Rapid review of multi-wave studies. Epidemiol. Psychiatr. Sci..

[5] Turner A.I., Smyth N., Hall S.J., Torres S.J., Hussein M., Jayasinghe S.U., Ball K., Clow A.J. (2020). Psychological stress reactivity and future health and disease outcomes: A systematic review of prospective evidence. Psychoneuroendocrinology.

[6] Lippi G., Henry B.M., Bovo C., Sanchis-Gomar F. (2019). Health risks and potential remedies during prolonged lockdowns for coronavirus disease 2019 (COVID-19). Diagnosis.

[7] Freiberg A., Schubert M., Starke K.R., Hegewald J., Seidler A. (2021). A Rapid Review on the Influence of COVID-19 Lockdown and Quarantine Measures on Modifiable Cardiovascular Risk Factors in the General Population. Int. J. Environ. Res. Public Health.

[8] Douglas M., Katikireddi S.V., Taulbut M., McKee M., McCartney G. (2020). Mitigating the wider health effects of COVID-19 pandemic response. BMJ.

[9] Bongard V., Dallongeville J., Arveiler M., Ruidavets J.-B., Amouyel P., Wagner A., Ferrières J. (2013). Attainment of low-density lipoprotein cholesterol target in the French general population according to levels of cardiovascular risk: Insights from the MONA LISA study. Arch. Cardiovasc. Dis..

[10] Bongard V., Arveiler D., Dallongeville J., Ruidavets J.-B., Wagner A., Simon C., Marécaux N., Ferrieres J. (2016). Food groups associated with a reduced risk of 15-year all-cause death. Eur. J. Clin. Nutr..

[11] Giovannelli J., Dallongeville J., Wagner A., Bongard V., Laillet B., Marecaux N., Ruidavets J.B., Haas B., Ferrieres J., Arveiler D. (2014). Validation of a Short, Qualitative Food Frequency Questionnaire in French Adults Participating in the MONA LISA-NUT Study 2005–2007. J. Acad. Nutr. Diet..

[12] Spitzer R.L., Kroenke K., Williams J.B.W., Löwe B. (2006). A Brief Measure for Assessing Generalized Anxiety Disorder. Arch. Intern. Med..

[13] Kroenke K., Spitzer R.L., Williams J.B.W. (2001). The PHQ-9. J. Gen. Intern. Med..

[14] Bu F., Bone J.K., Mitchell J.J., Steptoe A., Fancourt D. (2021). Longitudinal changes in physical activity during and after the first national lockdown due to the COVID-19 pandemic in England. Sci. Rep..

[15] Navarro-Pérez C.F., Fernández-Aparicio Á., González-Jiménez E., Montero-Alonso M.Á., Schmidt-RioValle J. (2021). Effects of COVID-19 lockdown on the dietary habits and lifestyle in a population in southern Spain: A cross-sectional questionnaire. Eur. J. Clin. Nutr..

[16] Fancourt D., Steptoe A., Bu F. (2021). Trajectories of anxiety and depressive symptoms during enforced isolation due to COVID-19 in England: A longitudinal observational study. Lancet Psychiatry.

[17] Saunders R., Buckman J.E.J., Fonagy P., Fancourt D. (2021). Understanding different trajectories of mental health across the general population during the COVID-19 pandemic. Psychol. Med..

[18] Caroppo E., Mazza M., Sannella A., Marano G., Avallone C., Claro A., Janiri D., Moccia L., Janiri L., Sani G. (2021). Will Nothing Be the Same Again? Changes in Lifestyle during COVID-19 Pandemic and Consequences on Mental Health. Int. J. Environ. Res. Public Health.

[19] González-Sanguino C., Ausín B., Castellanos M., Saiz J., Muñoz M. (2021). Mental health consequences of the COVID-19 outbreak in Spain. A longitudinal study of the alarm situation and return to the new normality. Prog. Neuro-Psychopharmacol. Biol. Psychiatry.

[20] Brülhart M., Klotzbücher V., Lalive R., Reich S.K. (2021). Mental health concerns during the COVID-19 pandemic as revealed by helpline calls. Nature.

[21] Xu Z., Fan J., Ding J., Feng X., Tao S., Zhou J., Qian L., Tao K., Hambly B.D., Bao S. (2021). The Impact of COVID-19 on Primary Care General Practice Consultations in a Teaching Hospital in Shanghai, China. Front. Med..

