# Supplementary material for: One-Year Impact of COVID-19 Lockdown-Related Factors on Cardiovascular Risk and Mental Health: A Population-Based Cohort Study
Source: Int J Environ Res Public Health. 2022 Feb 1;19(3):1684. doi: 10.3390/ijerph19031684 (PMC8835147; doi:10.3390/ijerph19031684)
Supplement: Supplementary file 1 [file ijerph-19-01684-s001.zip › FigureS1_GAD-7.pdf]

<u>GAD-7 Anxiety</u>

**Over the <u>last 2 weeks</u>, how often have you been bothered by the following problems?**<br>*(Use "✔" to indicate your answer"*	Not at all	Several days	More than half the days	Nearly every day
1. Feeling nervous, anxious or on edge	0	1	2	3
2. Not being able to stop or control worrying	0	1	2	3
3. Worrying too much about different things	0	1	2	3
4. Trouble relaxing	0	1	2	3
5. Being so restless that it is hard to sit still	0	1	2	3
6. Becoming easily annoyed or irritable	0	1	2	3
7. Feeling afraid as if something awful might happen	0	1	2	3

Column totals: ___ **+** ___ **+** ___ **+** ___

= ***Total Score*** _____
