# Supplementary material for: One-Year Impact of COVID-19 Lockdown-Related Factors on Cardiovascular Risk and Mental Health: A Population-Based Cohort Study
Source: Int J Environ Res Public Health. 2022 Feb 1;19(3):1684. doi: 10.3390/ijerph19031684 (PMC8835147; doi:10.3390/ijerph19031684)
Supplement: Supplementary file 1 [file ijerph-19-01684-s001.zip › FigureS2_PHQ-9.pdf]

PHQ-9 Depression

**Over the last 2 weeks, how often have you been bothered by any of the following problems?** *(Use "✔" to indicate your answer")*	**Not at all**	**Several days**	**More than half the days**	**Nearly every day**
1. Little interest or pleasure in doing things..................	0	1	2	3
2. Feeling down, depressed, or hopeless..................	0	1	2	3
3. Trouble falling or staying asleep, or sleeping too much...	0	1	2	3
4. Feeling tired or having little energy....................	0	1	2	3
5. Poor appetite or overeating................................	0	1	2	3
6. Feeling bad about yourself — or that you are a failure or have let yourself or your family down........................	0	1	2	3
7. Trouble concentrating on things, such as reading the newspaper or watching television..............................	0	1	2	3
8. Moving or speaking so slowly that other people could have noticed? Or the opposite — being so fidgety or restless that you have been moving .around a lot more than usual...	0	1	2	3
9. Thoughts that you would be better off dead or of hurting yourself in some way..	0	1	2	3
**Column totals**	___	+ ___	+ ____	+ ___

= ***Total Score*** _____
