# Supplementary material for: One-Year Impact of COVID-19 Lockdown-Related Factors on Cardiovascular Risk and Mental Health: A Population-Based Cohort Study
Source: Int J Environ Res Public Health. 2022 Feb 1;19(3):1684. doi: 10.3390/ijerph19031684 (PMC8835147; doi:10.3390/ijerph19031684)
Supplement: Supplementary file 1 [file ijerph-19-01684-s001.zip › Table S1.pdf]

Table S1: Food frequency questionnaire.

For each item, the subjects had to choose the frequency with which they consumed the food in question, from one of nine categories: "three times a day", "twice a day", "once a day", "four to six times a week", "two to three times a week ", "once a week", "once every two weeks", "once a month" and "never". These categories were then coded as a frequency of daily intake, with the following values: 3, 2, 1, 0.714, 0.357, 0.143, 0.071, 0.033 and 0.

Before lockdown, the FFQ concerned the usual intake during the last year.
During lockdown, the FFQ concerned the usual intake since the beginning of the lockdown.:
At 1-, 6- and 12-month evaluation, the FFQ concerned the usual intake since the last phone call.

	Three times a day	Twice a day	Once a day	Four to six times a week	Two to three times a week	Once a week	Once every two weeks	Once a month	Never
Poultry (chicken, turkey, duck, etc.)									
Meat (beef, pork, veal, lamb, rabbit, etc.)									
Deli meat (pâté, rillettes (potted meat), sausage, bacon, blood sausage, etc - but not ham)									
Ham (cooked, cured, smoked)									
Fish (fresh, frozen, canned, shellfish, etc.)									
Eggs									
Bread (sandwich, wholemeal, baguette, crispread, etc.)									
Fried food (fries, crisps, donuts)									
Carbohydrates (potatoes, pasta, rice, semolina, wheat...)									
Pulses (white and red beans, flageolet beans, lentils, split peas, chickpeas, broad beans, etc.)									
Raw or cooked vegetables and vegetable soups (green vegetables, carrots, tomatoes, excluding potatoes, carbohydrates and pulses)									

	Three times a day	Twice a day	Once a day	Four to six times a week	Two to three times a	Once a week	Once every two	Once a month	Never

					week		weeks		
Fruits (fresh, frozen, canned, compote, raw or cooked)									
Nuts (walnuts, hazelnuts, almonds, pistachios, peanuts etc.)									
Butter or low-fat butter (on toast or for cooking)									
Margarine or low-fat margarine (on toast or for cooking)									
Olive oil (seasoning or cooking)									
Other oils (seasoning, cooking, frying)									
Cheese (excluding fromage frais/cottage cheese)									
Milk									
Dairy products (yoghurts, fromage frais, cottage cheese)									
Other milk-based desserts (creams, custards, etc.)									
Other sweet desserts (pastries, pies, cakes, ice cream, etc.)									
Confectionary (candies, caramels, chocolate, cookies and biscuits, etc.)									
Breakfast cereal (corn flakes, muesli, etc.)									
