# Supplementary material for: One-Year Impact of COVID-19 Lockdown-Related Factors on Cardiovascular Risk and Mental Health: A Population-Based Cohort Study
Source: Int J Environ Res Public Health. 2022 Feb 1;19(3):1684. doi: 10.3390/ijerph19031684 (PMC8835147; doi:10.3390/ijerph19031684)
Supplement: Supplementary file 1 [file ijerph-19-01684-s001.zip › TableS2.pdf]

Table S2: Suspected risk factors and potential confounders (dependent variables) assessed (one by one) in univariate analyses for worsening cardiovascular risk, incident depression and anxiety outcomes during the 12-month post-lockdown follow-up

	Outcome						
**Dependent variables (suspected risk factors and potential confounders)**	**Increased antihypertensive, lipid-lowering or hypoglycaemic drug treatment**	**Decrease in physical activity ≥ 15 minutes/week**	**Weight gain > 2 kg**	**Decrease in diet quality [£]**	**Smoking ≥ 1 cigarette/day**	**Depression [PHQ-9>4]**	**Anxiety [GAD-7>4]**
Age (in years: 50 - 59 vs 60 - 69 vs 70 - 79 vs 80 - 89) - during lockdown	☒	☒	☒	☒[$]	☒[$]	☒[$]	☒
Gender (male vs female)	☒	☒[$]	☒	☒	☒	☒[$]	☒[$]
Number of persons living with participant - during lockdown	☒	☒[$]	☒		☒	☒[$]	☒[$]
Living with > 2 people - during lockdown [#]				☒[$]			
Home location (Urban vs Rural) - during lockdown	☒	☒[$]	☒	☒	☒[$]	☒[$]	☒
Educational level<br>< High school completion<br>≥ High school completion	☒[$]	☒	☒	☒[$]	☒[$]	☒	☒

Dependent variables (suspected risk factors and potential confounders)	Outcome						
	**Increased antihypertensive, lipid-lowering or hypoglycaemic drug treatment**	**Decrease in physical activity ≥ 15 minutes/week**	**Weight gain > 2 kg**	**Decrease in diet quality [£]**	**Smoking ≥ 1 cigarette/day**	**Depression [PHQ-9>4]**	**Anxiety [GAD-7>4]**
Professional activity - before lockdown<br>Not Working<br>Working<br>Retired	☒	☒	☒[$]	☒[$]	☒[$]	☒[$]	☒
Professional activity - during lockdown<br>Working (out of home) without in-person contact with the public (e.g. dustmen)<br>Working with in-person contact with the public (e.g. cashiers, nurses)<br>Teleworking<br>Not working	☒	☒	☒[$]	☒[$]	☒[$]	☒[$]	☒[$]
Change in bedtime / wake-up time ≥2h - during lockdown*	☒	☒	☒[$]	☒[$]	☒[$]	☒[$]	☒
Having more time for oneself ** - during lockdown	☒	☒	☒	☒[$]	☒	☒	☒

Dependent variables (suspected risk factors and potential confounders)	Outcome						
	Increased antihypertensive, lipid-lowering or hypoglycaemic drug treatment	Decrease in physical activity ≥ 15 minutes/week	Weight gain > 2 kg	Decrease in diet quality [£]	Smoking ≥ 1 cigarette/day	Depression [PHQ-9>4]	Anxiety [GAD-7>4]
During the last 7 days, lifestyle that didn’t suit the participant - during lock-down	☒[$]	☒[$]	☒[$]	☒	☒	☒	☒
Self-perceived risk of being contaminated by COVID-19 (on a scale from 1 to 10) - during lockdown	☒			☒	☒[$]	☒[$]	☒
Self-perceived risk of being contaminated by COVID-19 (on a scale from 1 to 10) ≥ 4 [median] - during lockdown [#]		☒[$]					
Self-perceived risk of being contaminated by COVID-19 (on a scale from 1 to 10) > 2 (first quartile) - during lockdown [#]			☒[$]				
Estimated relationship with partner (on a scale from 1 to 10) - during lockdown	☒	☒	☒	☒	☒		☒[$]

Dependent variables (suspected risk factors and potential confounders)	Outcome						
	Increased antihypertensive, lipid-lowering or hypoglycaemic drug treatment	Decrease in physical activity ≥ 15 minutes/week	Weight gain > 2 kg	Decrease in diet quality [£]	Smoking ≥ 1 cigarette/day	Depression [PHQ-9>4]	Anxiety [GAD-7>4]
Estimated relationship with partner (on a scale from 1 to 10) > 8 (median) - during lockdown [#]						☒[$]	
Worsening relationship with partner during lockdown	☒[$]	☒	☒	☒	☒[$]	☒	☒
History of high blood pressure - before lockdown	☒[$]	☒	☒	☒	☒[$]	☒	☒
History of hypercholesterolemia - before lockdown	☒[$]	☒	☒	☒	☒	☒	☒
History of diabetes - before lockdown	☒[$]	☒	☒	☒	☒	☒	☒
History of obesity - before lockdown	☒[$]	☒	☒[$]	☒	☒[$]	☒	☒

Dependent variables (suspected risk factors and potential confounders)	Outcome						
	Increased antihypertensive, lipid-lowering or hypoglycaemic drug treatment	Decrease in physical activity ≥ 15 minutes/week	Weight gain > 2 kg	Decrease in diet quality [£]	Smoking ≥ 1 cigarette/day	Depression [PHQ-9>4]	Anxiety [GAD-7>4]
History of CVD - before lockdown	☒[$]	☒	☒	☒[$]	☒	☒[$]	☒
Family history of premature coronary disease*** - before lockdown	☒	☒	☒	☒	☒	☒	☒
History of anxiety - before lockdown	☒	☒	☒	☒	☒	☒[$]	☒[$]
Anxiety drug treatment - before lockdown	☒	☒	☒	☒	☒	☒[$]	
History of depression - before lockdown	☒	☒	☒[$]	☒	☒	☒[$]	☒[$]
Depression drug treatment - before lockdown	☒	☒	☒	☒	☒		☒
Anxiety: GAD-7 - during lockdown	☒	☒	☒[$]	☒	☒	☒[$]	☒[$]
Anxiety (GAD-7>4) - during lockdown [#]						☒[$]	

Dependent variables (suspected risk factors and potential confounders)	Outcome						
	Increased antihypertensive, lipid-lowering or hypoglycaemic drug treatment	Decrease in physical activity ≥ 15 minutes/week	Weight gain > 2 kg	Decrease in diet quality [£]	Smoking ≥ 1 cigarette/day	Depression [PHQ-9>4]	Anxiety [GAD-7>4]
Depression: PHQ-9 - during lockdown				☒	☒	☒[$]	☒[$]
Moderate depression (PHQ-9≥10) - during lock-down [#]	☒[$]						
Depression (PHQ-9≥3 [median]) - during lockdown [#]		☒[$]					
Depression (PHQ-9>4) - during lockdown [#]			☒[$]				
Smoking - before lockdown<br>Yes, regularly (every day)<br>Yes, occasionally (<1cig/d)<br>No	☒	☒	☒[$]	☒[$]			☒

Dependent variables (suspected risk factors and potential confounders)	Outcome						
	Increased antihypertensive, lipid-lowering or hypoglycaemic drug treatment	Decrease in physical activity ≥ 15 minutes/week	Weight gain > 2 kg	Decrease in diet quality £	Smoking ≥ 1 cigarette/day	Depression [PHQ-9>4]	Anxiety [GAD-7>4]
Number of cigarettes/day - before lockdown	☒	☒	☒	☒			☒
Smoking (non-smokers vs < 3 cigarettes/day (ref) vs ≥ 3 cigarettes/day) - before lockdown #					☒$		
Smoking >2 cigarettes/day - before lockdown #						☒$	
Smoking - during lockdown<br>Yes, regularly (everyday)<br>Yes, occasionally (<1cig/d)<br>No	☒	☒	☒$	☒		☒	☒
Number of cigarettes/day - during lockdown	☒	☒	☒	☒		☒$	☒
Smoking - during lockdown	☒$	☒$	☒$	☒	☒$	☒	☒

Dependent variables (suspected risk factors and potential confounders)	Outcome						
	Increased antihypertensive, lipid-lowering or hypoglycaemic drug treatment	Decrease in physical activity ≥ 15 minutes/week	Weight gain > 2 kg	Decrease in diet quality [£]	Smoking ≥ 1 cigarette/day	Depression [PHQ-9>4]	Anxiety [GAD-7>4]
Decreased<br>No change<br>Increased							
Alcohol consumption - before lockdown<br>No<br>≥ 1 glass/week<br>≥ 1 glass/day	☒	☒		☒	☒	☒	☒[$]
Consumption ≥ 1 glass of alcohol/week - before lockdown			☒[$]				
Alcohol consumption (glasses/day) - before lockdown	☒	☒		☒	☒[$]	☒	☒
Alcohol consumption - during lockdown<br>No<br>≥ 1 glass/week<br>≥ 1 glass/day	☒		☒[$]		☒	☒	☒

Dependent variables (suspected risk factors and potential confounders)	Outcome						
	Increased antihypertensive, lipid-lowering or hypoglycaemic drug treatment	Decrease in physical activity ≥ 15 minutes/week	Weight gain > 2 kg	Decrease in diet quality [£]	Smoking ≥ 1 cigarette/day	Depression [PHQ-9>4]	Anxiety [GAD-7>4]
Consumption of ≥ 1 glass of alcohol /week - during lock-down				☒[$]			
Alcohol consumption (in glass/day) - during lockdown	☒		☒		☒	☒	☒
Consumption of > 3 glasses of alcohol/day - during lockdown [#]		☒[$]					
Alcohol consumption - during lockdown<br>Decreased<br>No change<br>Increased	☒	☒[$]	☒	☒	☒[$]	☒[$]	☒[$]
Physical activity (min/week) - before lockdown	☒[$]	☒[$]	☒	☒	☒[$]	☒	☒
Physical activity (min/week) - during lockdown	☒[$]	☒[$]	☒	☒	☒[$]	☒	☒

Dependent variables (suspected risk factors and potential confounders)	Outcome						
	Increased antihypertensive, lipid-lowering or hypoglycaemic drug treatment	Decrease in physical activity ≥ 15 minutes/week	Weight gain > 2 kg	Decrease in diet quality [£]	Smoking ≥ 1 cigarette/day	Depression [PHQ-9>4]	Anxiety [GAD-7>4]
Physical activity (min/week) - during lockdown<br>Decreased<br>No change<br>Increased	☒[$]	☒[$]	☒	☒	☒[$]	☒	☒
Housework**** (min/week) - before lockdown	☒	☒	☒	☒	☒	☒	☒
Housework**** (min/week) - during lockdown	☒	☒	☒	☒	☒	☒	☒
Housework**** (min/week) - during lockdown<br>Decreased<br>No change<br>Increased	☒[$]	☒	☒	☒[$]	☒	☒	☒
Increased screen time (hours/day) -	☒[$]	☒[$]	☒	☒[$]	☒[$]	☒	☒

Dependent variables (suspected risk factors and potential confounders)	Outcome						
	Increased antihypertensive, lipid-lowering or hypoglycaemic drug treatment	Decrease in physical activity ≥ 15 minutes/week	Weight gain > 2 kg	Decrease in diet quality £	Smoking ≥ 1 cigarette/day	Depression [PHQ-9>4]	Anxiety [GAD-7>4]
during lockdown							
Diet quality - during lockdown<br>Increased<br>No change<br>Decreased£	☒	☒	☒	☒	☒$	☒	☒$
Weight gain - during lockdown	☒	☒	☒$	☒	☒$	☒$	☒$

When the linearity hypothesis was not respected, continuous variables were transformed into ordinal variables using quartile distributions.

$ Variables associated with the endpoint of interest in univariate analyses with a P-value <.20 (corresponding to variables initially introduced into the multivariate analyses). * compared to pre-lockdown bedtime / wake-up time. ** As a positive effect of the lockdown felt by the participant. *** Family history of premature myocardial infarction, i.e. before 55 years old for the father or brother and before 65 years old for the mother or sister. **** Such as cleaning, tidying, do-it-yourself, gardening...

£ Decrease in diet quality was defined as in increase in consumption of sugary foods, alcohol, fat or carbohydrates, that was not compensated by increased fruit and vegetable, dairy (within a limit of 2.5 servings/day), or lean protein consumption. CVD: cardiovascular disease (ischemic heart disease, atherosclerotic cerebrovascular disease, atherosclerosis in other arteries such as aorta or lower limb arteries, chronic heart failure). GAD-7: Generalized Anxiety Disorder-7. PHQ-9: Patient Health Questionnaire-9. SD: Standard deviation. Ref: reference (Odds Ratio=1.00).Vs: versus.
